# Exploring chemical properties of essential oils from citrus peels using green solvent

**DOI:** 10.1016/j.heliyon.2024.e40088

**Published:** 2024-11-03

**Authors:** Katheryn L. Vasquez-Gomez, Diner Mori-Mestanza, Aline C. Caetano, Guillermo Idrogo-Vasquez, Carlos Culqui-Arce, Erick A. Auquiñivin-Silva, Efraín M. Castro-Alayo, Rosita Cruz-Lacerna, Harvey A. Perez-Ramos, César R. Balcázar-Zumaeta, Llisela Torrejón-Valqui, Cindy Yoplac-Collantes, Ives Yoplac, Segundo G. Chavez

**Affiliations:** a Instituto de Investigación, Innovación y Desarrollo para el Sector Agrario y Agroindustrial (IIDAA), Facultad de Ingeniería y Ciencias Agrarias, Universidad Nacional Toribio Rodríguez de Mendoza de Amazonas, Chachapoyas, 01001, Peru; b Instituto de Investigación para el Desarrollo Sustentable de Ceja de Selva (INDES-CES), Universidad Nacional Toribio Rodríguez de Mendoza de Amazonas, Chachapoyas, 01001, Peru; c Laboratorio de Nutrición Animal y Bromatología de alimentos, Facultad de Ingeniería Zootecnista, Agronegocios y Biotecnología, Universidad Nacional Toribio Rodríguez de Mendoza de Amazonas, Chachapoyas, 01001, Peru

**Keywords:** Cromatography, Spectroscopy, Volatile compounds, D-limonene, Raman

## Abstract

The research explored the chemical characteristics of essential oils (EOs) extracted from the peels of four citrus fruits grown in northeastern Peru (lime, sweet lemon, mandarin and orange). The essential oils were extracted by hydrodistillation using a green solvent, and subsequently, their physicochemical profile, bioactive, heat capacity, and RAMAN mapping were determined; in addition, the volatile composition was determined by gas chromatography (GC-MS), and the main phenols by liquid chromatography (UHPLC). The results evidenced that sweet lemon and mandarin essential oils had higher antioxidant activity (1592.38 and 1216.13 μmol TE/g) and total phenolic content (680.78 and 420.28 mg GAE/g). In contrast, sweet lemon peel essential oil had the highest total flavonoid content (23.18 mg QE/g). D-limonene was the most abundant aromatic compound in orange (>67 %), mandarin (>70 %), and sweet lemon (>72 %) EOs; however, in the lime, it was the lowest (37 %). The most abundant component was the cyclobutane, 1,2-bis(1-methylethylethylenyl)-, trans- (32 %).

## 1 Introduction

There is a growing demand for citrus in the food industry, where it is mainly consumed as fresh fruit [1]. However, due to processing, solid residues (especially peel, in a proportion of 55–60 %) are generated and are not efficiently utilized. Thus, citrus peel is considered a by-product with valuable applications [2] due to its rich composition in flavonoids, alkaloids, coumarins, limonoids (highly oxygenated triterpenoids), carotenoids, phenolic acids and antioxidants [[2], [3], [4], [5], [6]].

The potential of citrus peel lies mainly in essential oils (EOs) [7], which are characterized by their strong and pleasant aroma and are used in various foods, beverages, and pharmaceuticals [2,8]. To obtain EOs, there are different techniques; traditional ones, such as maceration, hydrodistillation, pressing and heating [9,10]; and assisted techniques, such as ultrasound, supercritical fluids and microwave [11,12], which have been reported in citrus studies [[13], [14], [15], [16]]. Lu et al. [7] mention that, due to the existence of oil sacs in citrus peel, EOs are generally extracted by pressing and distillation. The latter is more important due to the use of green solvents [9,17], which allows a higher extraction of EOs and isolation of phenolic and volatile compounds [18,19], being a "sustainable" and "green" method [2].

Due to the chemical composition of citrus peel EOs, they are the subject of constant studies, as they have beneficial compounds for health and are a new source for use in the food industry. For example, the abundant presence of D-limonene, γ-terpinene, α-terpineol, terpinen-4-ol, and citral has been highlighted [7,14,15,20]. Therefore, this study explored the characteristics of EOs from peels of four citrus fruits (lime, sweet lemon, mandarin, and orange) grown in the region of Amazonas, Peru; where the peel by-product is considered as a waste material. These fruits are characterized by their antioxidative properties and phenolic compounds [21,22].

Also, it was explored the physicochemical properties and individual components associated with the volatile and bioactive profile of EOs, such as D-limonene, coumarins, and psoralens [2,[23], [24], [25], [26], [27], [28]]. Therefore, in this study, chromatography and UV spectrophotometry were used [29], combined with Raman spectroscopy, which is characterized by rapid detection, low cost, and non-destructive nature [30]. This allowed us to explore the chemical properties of essential oils froms peels of our citrus fruits extracted with green solvent.

## 2 Materials and methods

### 2.1 Citrus fruit peel collection

**Fig. 1:**
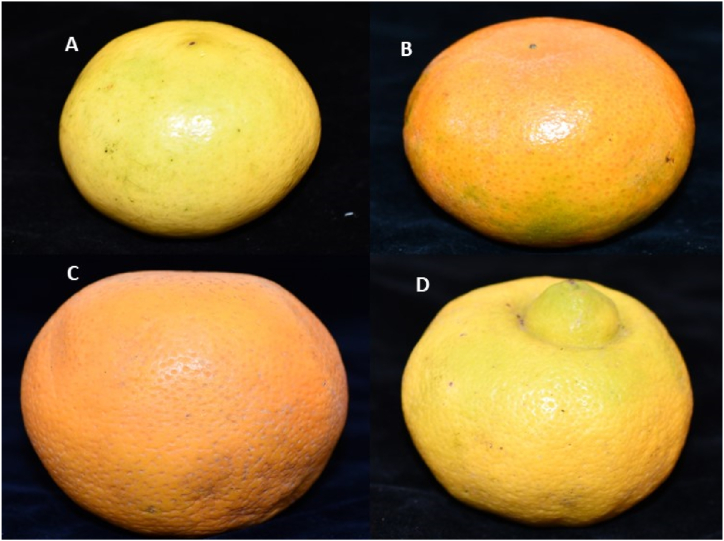
Citrus fruit is used to extract essential oils from peels. A: *Citrus limetta* sp.; B: *Citrus reticulata*; C: *Citrus sinensis*; D: *Citrus limetta* Risso.

Ripe fruits of sweet lemon (*Citrus limetta* sp. Fig. 1a), mandarin o tangerine (*Citrus reticulata*, Fig. 1b), orange (*Citrus sinensis*, Fig. 1c), and lime (*Citrus limetta* Risso, Fig. 1d) were obtained from plantations in the provinces of Utcubamba, Rodriguez de Mendoza and fruit juice stores in the city of Chachapoyas, Amazonas region, Peru (Fig. 1, and Fig. S1). The fruits were taken to the Engineering Laboratory of the Universidad Nacional Toribio Rodríguez de Mendoza de Amazonas for disinfection, conditioning, and obtaining the peels. Fresh peels were carefully removed, chopped (1 cm^2^), and kept at 4 °C.

### 2.2 Chemical reagents used

Folin-Ciocalteu reagent, gallic acid, quercetin, sodium acetate (CH_3_COONa), aluminum chloride, 2,2-diphenyl-1-picrylhydrazyl (DPPH), acetonitrile, 2,2′-azino-bis-3-ethylbenzothiazoline-6-sulfonic acid (ABTS+), potassium persulfate, Coumarin 72609- 100 mg (99. 9 %), Psoralen 89770-20 mg (≥98 %) (Sigma Aldrich, St. Louis, MO, USA), sodium carbonate (Spectrum Chemical Mfg. Corp, USA, 99.5 %), methanol (JT Baker, Deventer, The Netherlands).

### 2.3 Extraction of essential oils from peels

For the extraction of essential oils (EOs), the procedure of Phat et al. [31] was followed. The hydrodistillation was performed through a steam distiller (TECNAL, TE-2761, Brazil) at an extraction temperature of 80 °C. The distiller was started by adding 300 g of fruit peels with 500 ml of distilled water separately. The extraction time was approximately 1 h per sample. The water-essential oil mixture was then refrigerated at 1 °C to separate the oily from the aqueous phase. Then, the EOs was stored in amber glass bottles at 4 °C. The EOs yield was calculated according to Visakh et al. [15].

### 2.4 Antioxidant capacity by the DPPH (2,2-diphenyl-1-picrylhydrazyl) method

DPPH free radical scavenging activity was determined following the procedure by Guo et al. [32] and Smeriglio et al. [33]. An aliquot of 1 ml of essential oil (10 %) was mixed with 1 ml of 0.1 mM DPPH methanolic solution. The mixture was incubated in the dark at room temperature (≈ 22 °C) for 30 min. Then, in a spectrophotometer (EMC-11-UV Spectrophotometer) the absorbances at 517 nm (A_sample_) were obtained. In addition, the absorbance of a free-oil methanolic solution was obtained using the same procedure (A_white_). The measures were carried out in quadruplicate.

DPPH scavenging activity was determined using equation (1):

(1) $$\left(\%\;inhibition\right)=\left[\frac{\left(A_{white}-A_{sample}\right)}{A_{white}}\right]\times 100$$

### 2.5 Antioxidant capacity of essential oils with the ABTS ^+^ method

The procedure of Guo et al. [32] was followed with some modifications [34]. The radical cation ABTS^+^ was obtained by reacting 7 mM of aqueous ABTS^+^; then, 5 ml of 2.45 mM potassium persulfate was added to 88 μl of ABTS solution. The mixture was dark at room temperature (≈ 22 °C) for 12 h. The ABTS^+^ solution was diluted with ethanol (approximately 1:80) to obtain an absorbance of 0.700 ± 0.02 (734 nm). To measure the antioxidant activity of the EO, 30 μl of EO was added to 3000 μl of radical cation ABTS^+^. The absorbance was recorded twice, at time zero and after 30 min (when the absorbance was stabilized). To quantify the antioxidant activity of the essential oils, a curve was obtained with the Trolox standard (y = −0.0004x + 0.8502, R^2^ = 0.99) obtained from different concentrations (from 0 to 2000 mM).

### 2.6 Total phenolic content determination

Total phenolic content (TPC) was determined following the procedure of Singleton et al. [35] with some adjustments [33,36]. In brief, 0.5 ml of diluted essential oil (2.5 mg/0.5 ml ethanol) and 2.5 ml of diluted Folin-Ciocalteu's reagent (1:10 ultrapure water) were mixed for the reaction. Then, 2 ml of sodium carbonate solution (7.5 %) was added to the mixture, stirred vigorously, and reacted for 60 min. The absorbance was measured at 764 nm in a UV–Vis spectrophotometer (EMC-11-UV SPECTROPHOTOMETER). A standard curve for gallic acid (y = 0.0004x + 0.0212, R^2^ = 0.9986) with dilutions between 0 and 2500 mM was performed to quantify the total phenolic content. The result was expressed in mg gallic acid equivalents per gram of EO (mgGAE/g).

### 2.7 Total flavonoid content determination (TFC)

The method employed by Ndayishimiye et al. [36] was used. In brief, a mixture containing 0.5 ml of EO (diluted in 4.5 ml in ethanol), 0.5 ml of methanol, 50 μl of AlCl_3_ (10 %), 50 μl of 1 M potassium acetate, and 1.4 ml of distilled water was made. The mixture was left for 30 min at room temperature (≈ 22 °C). Afterward, the absorbance was recorded at 415 nm. The quantification of TFC was based on the quercetin standard curve (y = 0.0001x + 0.0119, R^2^ = 0.996) and expressed as mg quercetin equivalent (QE)/g EO.

### 2.8 Refractive index and density of essential oils

The refractive indices of essential oils extracted from citrus peels (orange, lime, mandarin, and sweet lemon) were measured using an ABBE-type refractometer (Brand, 2WAJ, USA). The instrument was thoroughly cleaned with distilled water and ethanol before each measurement. The n-D values were measured to calibrate the refractometer, and the accuracy of n^20^_D_ was ±0.0007. The density of the essential oils was determined (≈ 22 °C, room temperature) using the mass over volume formula [37,38].

### 2.9 Thermal properties of essential oils

The thermal properties of essential oils were measured using a differential scanning calorimeter DSC (TA Instruments, Discovery DSC 2500, New Castle, USA). The measurements were performed in a dynamic nitrogen atmosphere (50 ml/min). Each EO sample had a mass of approximately 10 mg. The samples were heated to a rising temperature from 25 up to 245 °C at a heating rate of 10 °C/min, as posited by Hosseini et al., Mohammadi et al., and Yang et al. [[39], [40], [41]]. This rising temperature provides insight into thermal transitions due to energy gain, which causes a separation between molecules [[42], [43], [44]]. Instrument control and data analysis were carried out using TRIOS software, which allowed the calculation of maximum and onset temperatures, enthalpy, and heat flux [37].

### 2.10 Raman spectral acquisition

Raman spectral acquisition of four essential oil samples from citrus peels were made using a Raman focal microscope system (Horiba Scientific, XploRA plus, Montpellier, France) [45]. We measured the spectra oil drop covered with a cover glass to protect it from evaporation, were obtained using a 532 nm laser as excitation light with a filter (50 %). Experimental conditions were: 100 nm slit width, 100 μm pinhole, x50/0.90 NA Vis-LWD air objective, and 1 s acquisition time with 2 accumulations. The Raman signal was obtained using a 600 lines/mm grating centered between 800 and 3100 cm^−1^. The acquired spectra were corrected from 1000 to 1800 cm^−1^, smoothed, and baseline corrected using LabSpec 6 Suite software [46].

### 2.11 Preparation of standards and samples of essential oils in gas chromatography

The (R)-(+)-Limonene and n—n-alkanes C 8 -C 20 standards and the essential oils were diluted in ethyl acetate solvent separately. The standard n—n-alkanes C 8 -C 20 was diluted in ethyl acetate (1:3) ml. The standard (R)-(+)-Limonene was diluted in different concentrations (1000, 100, 50, 25, 12.5,6.25 μg/ml), and the essential oils were diluted at 12000 μg/ml.

- *Volatile Profile: Characterization of volatile compounds in GC-MS*

The volatile compounds of the essential oil samples were analyzed through gas chromatography in an Agilent GC System model 7890B, coupled with the MSD 5977B quadrupole mass spectrometer. Analytical conditions are DB-5MS UI capillary column (60 m × 0.25 mm x 1.0 μm) and helium as carrier gas (1 ml/min). The injection will be performed in split mode (50:1), the injected volume is 1 μL (12000 μg/ml essential oil/C4 H 8 O 2 v/v), and the injector and detector temperature is 250 °C and 280 °C, respectively. The oven temperature was 60 °C for 6 min, then increased to 270 °C at 3 °C/min and held at 270 °C for 4 min. The ionization voltage is set to 70 eV, the electron multiplier to 900 V, and the ion source temperature to 230 °C. Mass spectral data are obtained in scan mode over a mass (*m*/*z*) range of 45–450 amu. The detected compounds are identified by comparing the mass spectra with the database of the National Institute of Standards and Technology (NIST Library 17) [34], for the identification of the compounds was confirmed by injection of the standard n-alkanes (C 8 -C 20) and the comparison of their retention rates [33].

### 2.12 Quantification of chemical compounds of essential oils by liquid chromatography

Essential oil samples were diluted with methanol (1–2 ml of sample in 1 ml of methanol) and centrifuged (30 min, 5000 rpm) before transferring to vials for analysis. For the characterization of phenolic compounds (coumarins and furanocoumarins), the procedure described by Cruz et al. [47], Balcázar-Zumaeta et al. [34], and Cortez et al. [48]. The injection volume of each solution was 10 μl in an ultra-high-performance liquid chromatography system (UHPLC, Agilent Technologies, 1290 Infinity II, Germany) equipped with a multisample (G7167B), flexible pump (G7104A), column oven (G7116B), diode array detector (G7117B), and a Zorbax Eclipse Plus C18 column (Agilent Technologies, USA) of 4.6 × 250 mm and 5 μm particle size. The mobile phase consisted of a linear gradient combining solvent A: 2 % acetic acid in water and solvent B: acetonitrile, water, and acetic acid (400:90:10 v/v/v/v).

The compounds (coumarin, psoralen and p-cumaric acid) were identified on a DAD detector set at 280 nm. The run time was 22 min, the temperature was 26 °C, and the flow rate was 0.75 ml/min. All standards used for quantitative determination were from Sigma-Aldrich, St.Louis, MO. The gradient program was 0–2 min, linear 90 % A and 10 % B; 2–5 min, linear 88 % A and 12%B; 5–8 min, linear 86 % A and 14%B, 8–10 min, 84 % A and 16%B; 10–12 min, linear 82 % A and 18%B; 12–22 min, 90 % A and 10%B. To quantify the phenolic compounds of the essential oils, standard curves were obtained for coumarin (y = 0.0000037x + 0.2154384), psoralen (y = 0.0000111x + 0.4174412), and p-coumaric acid (y = 0.00000518x + 0.07278492) at concentrations from 0 to 1 mg/ml.

### 2.13 Data analysis

To compare the EOs chemical properties, an analysis of variance was performed using the Tukey statistical test at the level (p < 0.05) and minimum significant differences; statistical analyses were performed using RMarkdown free software (RStudio, version 2022.07.2 + 576, Boston, MA, USA). A correlogram was constructed using Minitab 20 software. Spearman correlation was used to determine the correlation between the chemical properties of the essential oils.

## 3 Results and discussion

### 3.1 Yield of essential oils obtained from citrus peels

The results indicated that a higher yield of 1.0 % was obtained from citrus peels in *C. sinensis*, values that were comparable to those previously reported by Brahmi et al., Luro et al., and Singh et al. [13,49,50]. In contrast, the yield of essential oil extracted from *C. limett*a Risso was 0.66 %, a value within the range reported by Jayagoudar et al. and Singh et al. [51,52]. Similarly, the yield (0.5 %) in *C. reticulata* was previously reported (0.46–1.75 %) [49]. Ultimately, the yield of *C. limetta* sp. (0.16 %) was below what Arafat et al. [53] reported. It should be noted that the yields reported by Arafat et al. [53] are moderate and dependent on factors such as maturity, provenance, and growing conditions [54]. Additionally, the variety and thickness of the peel are believed to be contributing factors, as they influence the moisture content of the peel (in citrus peel, according to previous studies [55,56], the moisture between 75 and 90 %), which is dependent on its spongy quality and capacity to absorb oils [57,58].

### 3.2 Antioxidant capacity of citrus peel essential oil

**Fig. 2:**
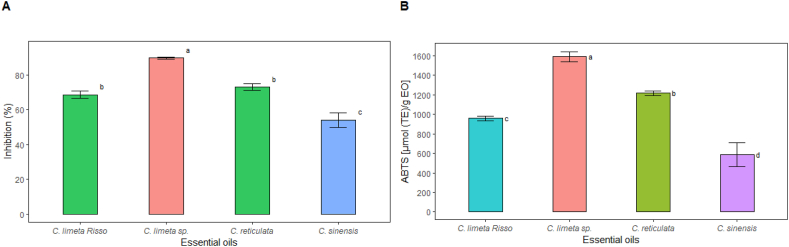
Antioxidant capacity of essential oils of four citrus fruits by (a) DPPH method and (b) ABTS method. Bars indicate standard deviation (n = 3). Different letters indicate statistically different groups between essential oil types by Tukey's test (sig.≤0.05).

**Fig. 3:**
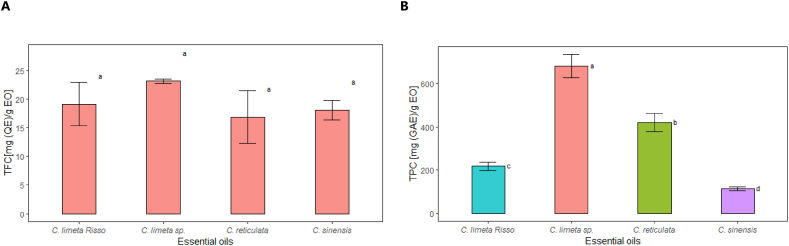
(a) Total flavonoid content (TFC), and (b) Total phenolic content (TPC) of four citrus peel essential oils. Bars indicate standard deviation (n = 3). Different letters indicate statistically different groups between essential oil types with Tukey's test (sig.≤0.05).

Essential oils are a rich source of antioxidants [59]. As illustrated in Fig. 2, the essential oil of *C. limetta* sp. exhibits a higher antioxidant capacity than the other oils due to the high availability of phenolic compounds and flavonoids. When considered together, Fig. 2, Fig. 3 demonstrate that these compounds enhance the antioxidant capacity of the essential oil [60,61]. In addition, there is a slight difference between the techniques used. However, it can be observed that *C. reticulata* EO is the second in antioxidant power *in vitro*, and orange essential oil has the least antioxidant capacity (p < 0.05).

Numerous antioxidants are commercially available for use in foods; however, natural rather than synthetic antioxidants are better because of their safety, functional, and sensory properties [62]. The data obtained with higher values of antioxidant capacity in both methods as DPPH and ABTS+ were 89.63 % in the EO extracted from sweet lemon peel and 1592.38 (μmol TE/g of EO) in the EO from sweet lemon peel, respectively. The differences observed with the two techniques are due to the reaction specificity [63]. By the DPPH method, the EO extracted from lime peel was 68.63 % and 54 % from orange (Fig. 2a). In contrast, in the ABTS + method, the same results were obtained with the lime peel EO (959.25 μmol TE/g of EO) containing better antioxidant capacity compared to the orange peel EO (586.75 μmol TE/g of EO) (Fig. 2b). In both methods, it can be seen that the EO extracted from lime peel showed higher antioxidant capacity as opposed to orange, which could be due to the higher content of D-limonene and linalool in the EO from the peel of this fruit. On the other hand, antioxidant activity values higher than those reported for Navel orange (44.45 % DPPH) were obtained [13]. Also, the antioxidant capacity of the four studied EOs was higher than those reported by other authors for EOs of similar fruits [32,50]. Higher antioxidant capacity values in this type of essential oil have been related to high contents of γ-terpinene, terpinolene, geraniol, β-pinene, and myrcene [64], which could allow us to suppose that the EOs of citrus fruits grown in Amazonas, Peru, are rich in these compounds.

### 3.3 Total flavonoid and phenolic content

Although the flavonoid content of the four essential oils ranged between 18.92 and 23.18 mg QE/g (Fig. 3a), these secondary metabolites have been identified in citrus peel [65], with approximately 117 flavonoid species demonstrated to reduce oxidative stress and exhibit anti-inflammatory and neuroprotective properties [66]. The combined presence of flavonoids and phenolic compounds results in a higher antioxidant efficacy, as both types of compounds can act on different oxidation mechanisms and contribute against oxidative stress [67,68]. No significant differences were found among them (p > 0.05); consequently, these EOs are very similar in total flavonoid content. In Fig. 3, following the trend observed in the antioxidant capacity (Fig. 2) of the EOs studied, *C. limetta* sp. had the highest total phenolic content (680.77 mg GAE/g, Fig. 3b), followed by the EOs of *C. reticulata*, *C. limetta* Risso and *C. sinensis*.

Significant differences were also observed between the total phenolic content of the EOs for the four fruit peels but not in the antioxidant capacity, indicating the presence of other non-phenolic compounds in the EOs. On the other hand, mandarin peel EO had 420.28 mg GAE/g lower values than previous works (40.94 mg GAE/g) [33,36]. However, sweet lemon peel EO had high phenolic and flavonoid content (23.18 mg QE/g EO), as reported by Maurya et al. [29].

### 3.4 Refractive index (RI) and density of essential oils

The refractive index and density values of the EOs studied are similar for the four fruits, as shown in Table 1. The refractive index value is highly dependent on the density and extraction time, as it indicates the change in the composition of each sample. The essential oils of citrus peels had a refractive index ranging between 1.468 and 1.475. It has been found that the lower the refractive index, the lighter the compounds in the sample, such as terpenes. Similarly, a higher refractive index indicates heavier compounds, such as oxygenates [69].

**Table 1:** Refractive index and density values of the four types of citrus peel essential oils.

EO type	RI	Density (g/ml)
*C*. *sinensis*	1.475^a^	0.9854^d^
*C*. *limetta* Risso	1.468^d^	1.0022^a^
*C*. *reticulata*	1.472^b^	0.9932^c^
*C*. *limetta* sp.	1.470^c^	0.9972^b^

The essential oils showed a yellow-orange hue associated with the chemical composition of EOs obtained [70,71], which is due to the presence of oxygenated monoterpenes according to Singh et al. [50]. These characteristics align with those previously documented by Ling et al. [72], serving as a qualitative indicator of the EOs high purity [72,73].

### 3.5 Thermal behavior of the essential oils (DSC)

Table 2 shows the data of the four essential oils' differential scanning calorimetry (DSC), the onset temperature is the temperature at which the transition begins, and the maximum temperature is the temperature at which the transition is completed [74]. The essential oil of *C. sinensis* peels presented higher enthalpy (153.05 J/g) than the other three essential oils. However, the essential oil of *C. limetta* Risso skin had a tighter onset temperature and maximum temperature (58.38 and 118.7 °C, respectively) compared to the other essential oils, where achieving high temperatures indicates favorable thermal stability [75]. The onset temperature in the calorimetric profile of EOs is an indicator of their oxidative susceptibility. Samples with high initiation temperatures have the highest stability. Similarly, the heating rate is another indicator of susceptibility [76]. The essential oil of *C. limetta* Risso has higher oxidative stability since its onset temperature was the highest (58.38 °C), followed by the EOs of *C. sinensis* (49.44 °C), *C. limetta* sp. (38.80 °C) and *C. reticulata* (26.19 °C). Variations in the content of EOs' volatile compounds influence their thermal behavior. The interaction of these compounds with others in the EO matrix can affect their performance, heat retention, and aroma release [75]. Therefore, the results in this study are necessary to ensure the thermal compatibility of these essential oils with other ingredients, avoiding undesired reactions during the manufacturing and storage of the final product [76,77].

**Table 2:** Differential scanning calorimetry data of essential oils.

Muestras	Samples Heat flux (W/g)	Initial temperature (°C)	Max temperature (°C)	Enthalpy (J/g)
*C*. *sinensis*	−0.798	49.44	94.97	153.05^a^
*C*. *limetta* Risso	−0.485	58.38	118.7	130.78^a^
*C*. *limetta* sp.	−0.287	38.80	94.18	87.507^b^
*C*. *reticulata*	−0.015	26.19	91.67	5.4116^c^

Different letters indicate statistically different groups between essential oil types by Tukey's test (sig.≤0.05).

### 3.6 Quantification of phenolic compounds (coumarin, psoralen, and p-cumaric acid) in UHPLC

The four citrus species contain coumarins in different concentrations, where the essential oil of *C. limetta* Risso had the highest concentration of this compound. Psoralen is a compound that is part of the furanocoumarins group; this compound was found only in *C. limetta* Risso and *C. reticulata*, while p-coumaric acid was only found in *C. reticulata* (Table 3). As Mahato et al. [78] reported, coumarins and furanocoumarins protect plants against fungal infections. According to Fan et al. [79], citrus peel EOs contain these compounds. In this study, *C. limetta* Risso and *C. reticulata* EO contain coumarins. In *C. reticulata* EO, p-coumaric acid was identified and quantified, indicating that these essential oils could be used as natural antifungal agents.

**Table 3:** Content of main phenolic compounds of essential oils extracted from the peels of four citrus fruits (mg/ml).

EO type	Coumarin (RT^a^: 11.14)	Psoralen (RT: 16.63)	p-cumaric acid
*C*. *limetta* sp.	0.28 ± 0.00025^c^	ND	ND
*C*. *sinensis*	0.37 ± 0.00284^b^	ND	ND
*C*. *limetta* Risso	0.40 ± 0.00071^a^	0.84 ± 0.00033^a^	ND
*C*. *reticulata*	0.29 ± 0.00632^c^	0.63 ± 0.00042^b^	0.073 ± 0.000002^a^

^a^RT: Retention time.

### 3.7 Correlation between phenolic compounds and essential oil characteristics

**Fig. 4:**
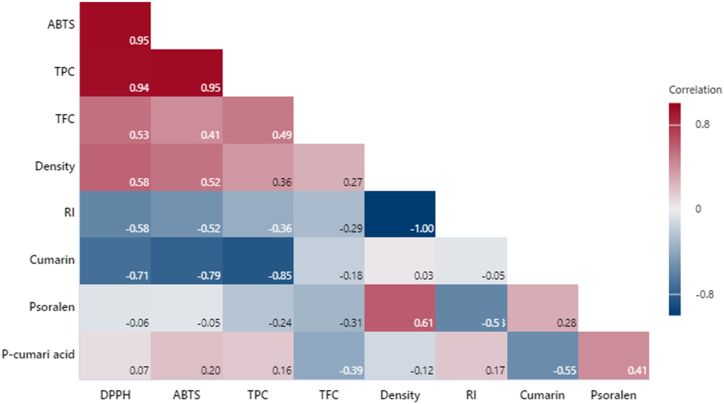
Correlation analysis of essential oil chemical properties.

In Fig. 4, as expected, the variables associated with the antioxidant capacity of the essential oils are highly correlated (ABTS, DPPH, and TPC; p < 0.05). The refractive index is inversely correlated with the density of essential oils (R = −1). Also, coumarin content is inversely correlated with antioxidant capacity (p < 0.05). Finally, high psoralen content is associated with higher density and higher antioxidant activity of the essential oils (p < 0.05), confirming what has been demonstrated by previous similar works [[23], [24], [25]].

### 3.8 Quantification of volatile compounds of essential oils by GC-MS

**Fig. 5:**
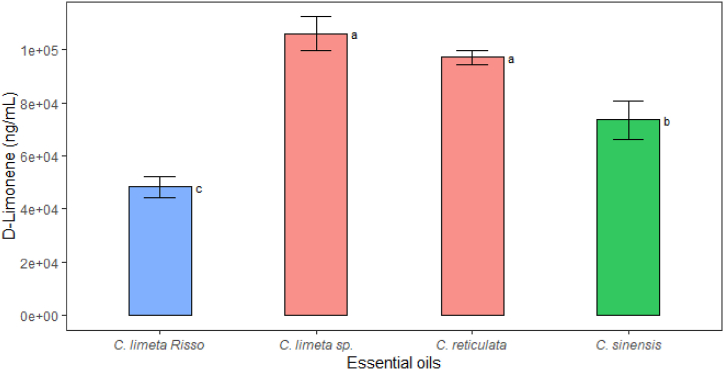
Concentration D-Limonene in essential oils by GC-MS. Bars indicate standard deviation (n = 3). Different letters indicate statistically different groups between essential oil types with Tukey's test (sig.≤0.05).

The essential oils of the four citrus species contained D-limonene in different concentrations; the essential oil of *C. limetta* sp. is the one with the highest concentration of this volatile compound [20]. According to Ref. [80], D-limonene is the main component of the essential oil of citrus fruits such as *C. sinesis* (84.75 %), *C. reticulata* (83.65 %), *C. maxima* (87.54 %), lemon (36.70 %), *C. medica* redonda (71.98 %) and *C. medica* oblonga (65.13 %); values that agree with those found in this research (*C. limetta* sp with 72.52 %, *C. reticulata* with 70.99 %, *C. sinensis* with 67.72 % and *C. limetta* Risso with 37.05 %). The presence of this compound is responsible for the fragrance and potential use in the food industry [72]. As illustrated in Fig. 5, the *C. limetta* sp. and *C. reticulata* exhibited elevated levels of D-limonene, a highly lipophilic cyclic monoterpene characteristic of citrus fruits. This compound serves as a natural preservative in food, as evidenced by the findings of Ali et al., Fisher & Phillips, Negro et al., and Ruiz & Flotats [[81], [82], [83], [84]].

**Fig. 6:**
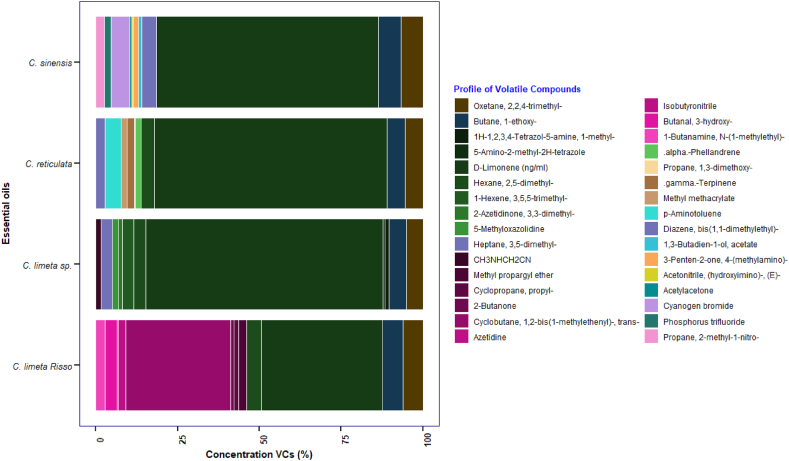
Concentrations (%) of volatile compounds identified by GC-MS in essential oils.

The number of volatile compounds identified (Fig. 6) in this study is higher than reported by Tunç & Odabaş [14]. The essential oils of citrus peels were evaluated, and the predominant compound was D-limonene, followed by cyclobutane, 1,2-bis(1-methylethenyl)-trans, butane, 1-ethoxy, and γ-ter. Compared to previous studies on lemon essential oil, pinene (antioxidant) and alpha-phellandrene (antimicrobial agent) were the predominant components, with D-limonene ranging between 60 % and 70 % of the total composition [26]. The remaining compounds identified were β-pinene, γ-terpinene, α-pinene, sabinene, myrcene, α-tuyene, and others [61].

Table 4 reveals the presence of 32 volatile compounds, with a notable prevalence of monoterpenes, comprising 85 %–95 % of the lemon essential oil. The most active aromatic chemical component was identified as citral, with a concentration range of 1.8 %–2.5 %. Other active aromatic compounds have been identified, including neryl acetate, geranyl acetate, and citronellal. The aromatic characteristics of lemons can vary depending on the variety.

**Table 4:** Relative abundance (%) of volatile compounds of essential oils.

RT (min)	Compound name	RI	LRI	*C. sinensis*	*C. limetta* Risso	*C. reticulata*	*C. limetta* sp.
	**Monoterpenes**						

26.91	D-Limonene	1041	1018	67.72	37.05	70.99	72.52
28.37	.alpha.-Phellandrene	1068	1005	ND	ND	1.83	ND
28.37	.gamma.-Terpinene	1068	1060	ND	ND	2.24	ND

	**Ethers**						

9.17	Methyl propargyl ether	706	493	ND	2.42	ND	ND

	**Esters**						

9.17	Methyl methacrylate	706	714	ND	ND	1.78	ND

	**Alkanes**						

9.54	Butane, 1-ethoxy-	713	669	7.08	6.2	5.68	5.25
9.55	Oxetane, 2,2,4-trimethyl-	713	664	6.6	6.09	5.32	5
9.56	Propane, 1,3-dimethoxy-	713	699	ND	ND	0.2	ND
10.97	Cyclopropane, propyl-	741	609	ND	1.46	ND	ND
10.97	Hexane, 2,5-dimethyl-			ND	4.48	4.19	3.8
10.97	Heptane, 3,5-dimethyl-			ND	ND	4.62	3.34
26.91	Cyclobutane, 1,2-bis(1-methylethenyl)-, trans-	1041	934	ND	31.99	ND	ND

	**Alkene**						

10.97	1-Hexene, 3,5,5-trimethyl-	741	769	ND	ND	ND	3.47
10.97	Diazene, bis(1,1-dimethylethyl)-	741	817	ND	ND	2.82	ND

	**Ketones**						

9.17	Acetylacetone	706	783	0.55	ND	ND	ND
9.18	2-Butanone	706	598	ND	1.15	ND	ND
10.97	1,3-Butadien-1-ol, acetate	741	783	1.02	ND	ND	ND
10.97	2-Azetidinone, 3,3-dimethyl-	741	778	ND	ND	ND	1.08

	**Aldehydes**						

9.17	Butanal, 3-hydroxy-	706	770	ND	3.67	ND	ND

	**Nitrogen compounds**						

10.97	Propane, 2-methyl-1-nitro-	741	736	2.62	ND	ND	ND

	**Amina**						

9.17	Isobutyronitrile	706	626	ND	0.49	ND	ND
9.17	2-(methylamino)acetonitrile	706	763	ND	ND	ND	1.65
9.18	1-Butanamine, N-(1-methylethyl)-	706	795	ND	2.79	ND	ND
9.18	5-Methyloxazolidine	706	795	ND	ND	ND	1.95
9.55	Azetidine	713	625	ND	2.21	ND	ND
10.96	5-Amino-2-methyl-2H-tetrazole	741		ND	ND	ND	0.63
10.96	Acetonitrile, (hydroxyimino)-, (E)-	741	933	0.49	ND	ND	ND
10.97	1H-1,2,3,4-Tetrazol-5-amine, 1-methyl-	741		ND	ND	ND	1.3
10.98	3-Penten-2-one, 4-(methylamino)-	741	937	1.61	ND	ND	ND
26.91	p-Aminotoluene	1041	1072	ND	ND	4.93	ND

	**Inorganic**						

26.90	Cyanogen bromide	1041		5.78	ND	ND	ND
9.17	Phosphorus trifluoride	706		1.91	ND	ND	ND

∗RT: retention time; ND: non detected.

Additionally, the essential oil of lime was found to have a lower concentration of the compound D-limonene, with a percentage of 46.03 %, which aligns with the findings presented in Table 4. Reports reveal that numerous studies have reported similar results on the essential oils of citrus peels. For instance, the essential oil of the peel of *C. reticulata* has been found to contain D-limonene as the dominant component, representing 86.4 % of the total oil composition. In the aforementioned study, the concentration of D-limonene was only 70.99 %, a result that is consistent with the findings of previous research. D-limonene is one of three isomeric monoterpenes that differ in their two double bonds' positions (alpha- and beta-terpinene). The concentration of this compound and other chemical compounds among citrus can be attributed to some factors, including the specific citrus variety, the region in which it is grown, local environmental conditions, genotypes, and the time of year during which it is harvested [85].

### 3.9 Raman spectroscopy of essential oils

Table 5 illustrates the Raman spectra of the analyzed essential oils (EOs) in the region between 200 and 3200 cm^−1^. The peaks identified result from molecular vibrations and chemical functional groups' stretching modes (see Fig. S2). Peaks attributed to single C-C bond stretching vibrations of alkanes were identified in the 800–1200 cm⁻^1^ region, and stretching vibrations are the origin of the oscillation of all C-C lines in ∼1000–1200 cm-1 range according to Novikov et al. [86] and Streletskiy et al. [87]. The 1300–1700 cm⁻^1^ region encompasses double bonds (C=C) of terpenoids, methyl (CH), and hydroxyl (OH) groups of phenols, as observed in other studies [88]. Similarly, in the region between 2000 and 3200 cm^−1^, carbonyl groups (C=O) were identified [89], characteristic of carboxylic acids, aldehydes, and ketones. The absorption peak at 3014 cm^−1^ was attributed to the = C-H molecule, associated with CH stretching vibrations of aromatics, and was considered D-limonene. Furthermore, D-limonene exhibits a = CH bending vibration at 886 cm^−1^ [72]. For the essential oils (EOs), this vibration was identified for *C. sinensis* (893.32 cm^−1^). The presence of limetta Risso (891.17 cm^−1^) and *C. limetta* sp. (897.61 cm^−1^), as well as the skins of *C. reticulata* (892.21 cm^−1^), is indicative of the presence of this compound (Table 4). The low-wavenumber Raman peaks between 260 and 767 cm-1 refer to low-energy vibrational modes such as torsion and bending; they reflect molecular interactions and the chemical structure of the compounds in the EOs [90].

**Table 5:** Raman spectra of essential oils.

Link type^a^	Citrus essential oil source			
	*C. sinensis*	C. limetta sp.	*C. reticulata*	*C. limetta* Risso
			264.18	
			283.54	
	324.41	320.11	322.25	315.82
		343.73	343.76	
	442.48	438.19	438.39	442.48
	496.16	468.25	481.41	478.98
	539.09		502.92	547.68
			548.08	
		534.8	599.7	
	646.43	639.99	647.02	646.43
	706.55			712.99
δ(C–H)	766.66	770.95	767.46	766.66
υ(C–H)	803.15	809.59	808.33	805.3
υ(C–H)	893.32	897.61	892.21	891.17
δ(=C–H)	923.38	931.96		923.38
υ(C-C)	1028.57	1035.01	1014.8	1019.98
υ(C–C)	1082.24	1084.39	1087.93	1084.39
υ(C-C)	1116.59	1127.32	1126.64	1118.74
υ(C-C)	1157.38	1165.97	1163.2	1161.67
υ(C-O)	1213.2	1223.93		
	1245.4			
υ(C -H)	1303.37	1311.95	1309.45	1303.37
υ(OH)	1378.5	1384.95	1378.28	1384.95
υ(C -H)	1442.91	1447.2	1444.95	1445.06
υ(C -H)			1550.34	
			1580.45	
C = C	1651.15	1651.15	1651.43	1649.01
C = C	1683.36	1683.36	1683.69	1681.21
ν(H–C=O)				2731.01
υ(C -H)	2887.73	2892.02	2883.82	2892.02
δ(=C–H)	2919.93	2922.08	2920.38	2924.23
υ(C -H)	2969.31	2975.75	2974.15	2975.75
= C-H				3014.39
ν(H–C=C)			3088.14	3089.53

υ(C-C): Stretching vibrations [87]; υ (C -H): Methyl group; (C = C): Double bond group; (C = O): Carbonyl group; (H - C = O): Aldehydes; υ(C-O): Ester; υ(OH): Stretching vibrations and (δ) (=C–H): Bending vibrations associated with methyl group [91,92].

a Assignments according to references cited in section 3.6.

Regarding the possible composition of the EOs, as shown in the Raman spectrum studied (800 -1200 cm^−1^), there are seven Raman peaks for the skin EOs of *C. sinensis*, *C. limetta* Risso and *C. limetta* sp., while for the skin EO of *C. reticulata*, only 6 peaks were found, indicating that it differs from the other three studied EOs, which are characteristic of the C-C skeleton. The spectra in the mentioned region agree with some investigations already carried out on peony seed essential oil, with four Raman peaks of the C-C skeleton [93].

Considering that D-limonene has bending vibration = CH at 886 cm^−1^ for the di-substituted double bond [94], the peaks obtained for the EO of *C. sinensis* (893.32 cm^−1^), *C. limetta* Risso (891.17 cm^−1^) and *C. limetta* sp. (897.61 cm^−1^), and *C. reticulata* (892.21 cm^−1^) skins, are indicative of the presence of this compound. The two strong bands at 1680-1650 cm^−1^ are attributed to the C=C stretching modes. Two bands are expected for C=C stretching because D-limonene has two double bonds in its chemical structure. The strong and broad band centered at ∼1433 cm^−1^ is assigned to the CH_3_/CH^2^ bending mode, while the strong signal at 766 and 809 cm −1 can be attributed to a ring deformation mode of D-limonene [95].

The variation of the line position is related to the variation of the conjugation length as (-CX=CY-)n bonds. These bonds are associated with light absorption and spectral line positions. As n increases, the system exhibits increased electron localization. This leads to changes in vibrational modes, resulting in relatively broad or narrow lines at lower or higher frequencies, depending on the change in molecular vibrations [87]. Chemical and structural variations in EOs, such as the presence of different terpenes or aldehydes, can affect the degree of conjugation and thus change the positions of the Raman lines [96].

## 4 Conclusions

The essential oils derived from citrus peels are widely regarded as a cost-effective and sustainable alternative because environmental impacts are reduced by using biodegradable by-products, containing a high concentration of bioactive compounds, and are, therefore, an attractive option for application in various industries. The results of the GC-MS analysis demonstrated that the essential oils derived from the peels of *Citrus sinensis*, *Citrus limetta* Risso, *Citrus reticulata* and *Citrus limetta* sp. contain a diverse range of bioactive compounds that contribute to their distinctive properties. The principal component is D-limonene, constituting between 37.05 and 72.52 % of the mixture. This compound offers many biological advantages, including antioxidant, anti-inflammatory, antimicrobial and anticancer properties.

The essential oils demonstrated antioxidant activity, with two in particular, *C. limetta* sp. and *C. reticulata*, exhibiting notable results in DPPH analysis (89.63 %; 72.92 %), ABTS+ (1592.38 μmol TE/g; 1216.13 μmol TE/g). The total flavonoid content was found to be 1216.13 μmol TE/g, while the total polyphenol capacity was 680.78 mg GAE/g and 420.28 mg GAE/g, respectively. Similarly, the analysis of the most representative non-volatile compounds in the EOs revealed the presence of coumarins, psoralen and p-coumaric acid, which have been demonstrated to possess biological activities such as antibacterial properties. The chemical properties of these essential oils, extracted from the peels of the four types of citrus fruit, have been analyzed and quantified. It is evident that they are an excellent source of bioactive and aromatic compounds that can be used in the food industry to develop potentially functional foods.

## CRediT authorship contribution statement

**Katheryn L. Vasquez-Gomez:** Writing – review & editing, Investigation, Conceptualization. **Diner Mori-Mestanza:** Writing – original draft, Visualization, Investigation, Funding acquisition, Conceptualization. **Aline C. Caetano:** Validation, Investigation, Data curation. **Guillermo Idrogo-Vasquez:** Funding acquisition. **Carlos Culqui-Arce:** Methodology. **Erick A. Auquiñivin-Silva:** Formal analysis. **Efraín M. Castro-Alayo:** Supervision, Software, Resources, Formal analysis. **Rosita Cruz-Lacerna:** Project administration. **Harvey A. Perez-Ramos:** Methodology. **César R. Balcázar-Zumaeta:** Writing – review & editing, Validation, Software. **Llisela Torrejón-Valqui:** Methodology, Data curation. **Cindy Yoplac-Collantes:** Methodology. **Ives Yoplac:** Data curation. **Segundo G. Chavez:** Writing – original draft, Resources, Investigation.

## Data availability statement

Data will be made available on request.

## Funding statement

The APC was financed by Vicerrectorado de Investigación - Universidad Nacional Toribio Rodríguez de Mendoza de Amazonas. This research was funded by the Consejo Nacional de Ciencia, Tecnología e Innovación Tecnológica-Concytec (Project: Nanoencapsulación de aceites esenciales de cáscaras de cítricos extraídos con disolventes verdes para desarrollar chocolates finos de aroma potencialmente funcionales, contract Nº PE501080039-2022-PROCIENCIA) of the Peruvian Government.

## Declaration of competing interest

The authors declare that they have no known competing financial interests or personal relationships that could have appeared to influence the work reported in this paper.
