# Supplementary material for: Exploring chemical properties of essential oils from citrus peels using green solvent
Source: Heliyon. 2024 Nov 3;10(21):e40088. doi: 10.1016/j.heliyon.2024.e40088 (PMC11570516; doi:10.1016/j.heliyon.2024.e40088)
Supplement: Multimedia component 1 [file mmc1.docx]

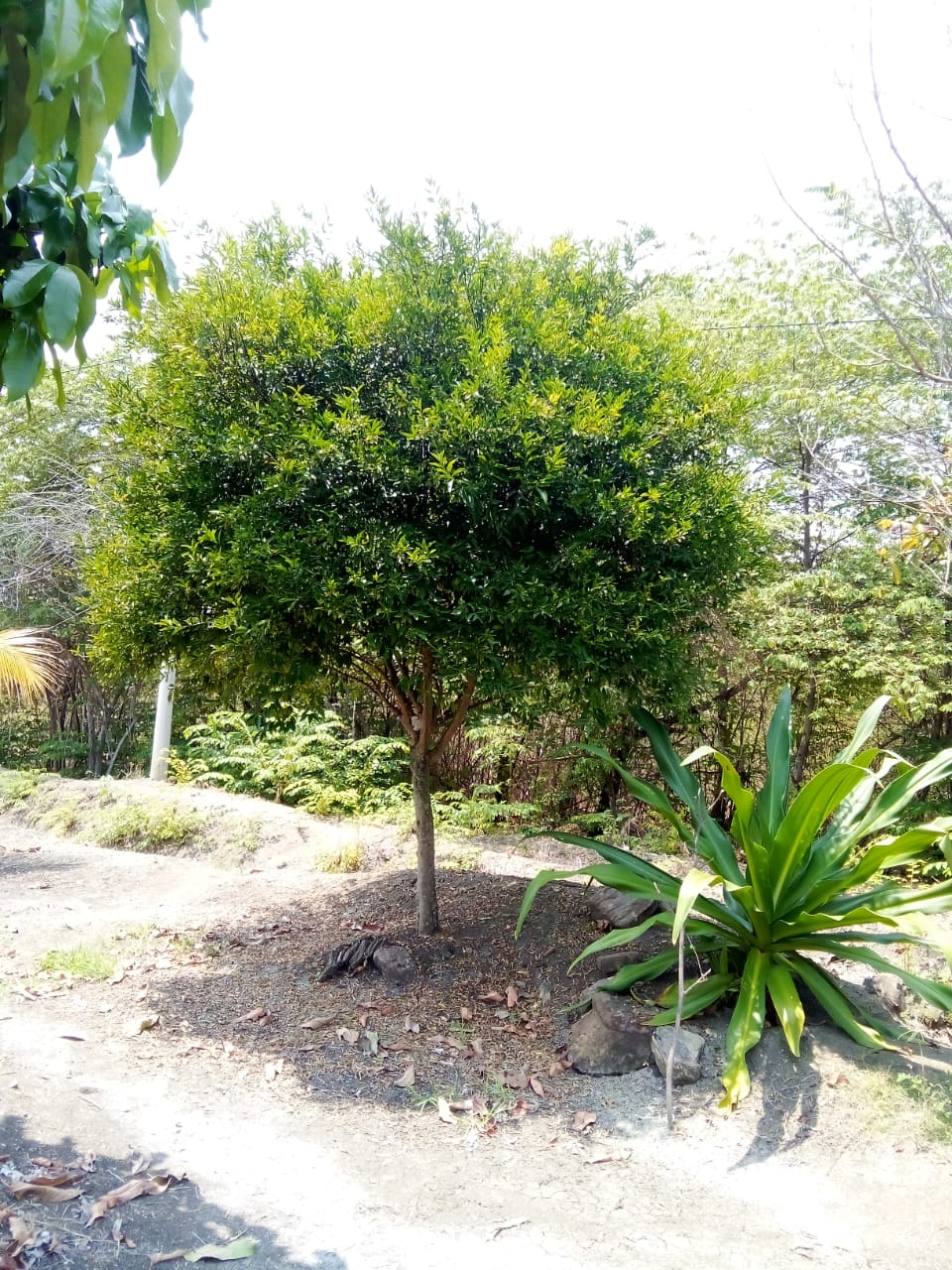


**Figure S1 (a).** *Citrus reticulata* tree (mandarin)

Location: Utcubamba (Jamalca; 1184 m.a.s.l,; 5° 53' 39'' S; 78° 14' 18'' W)


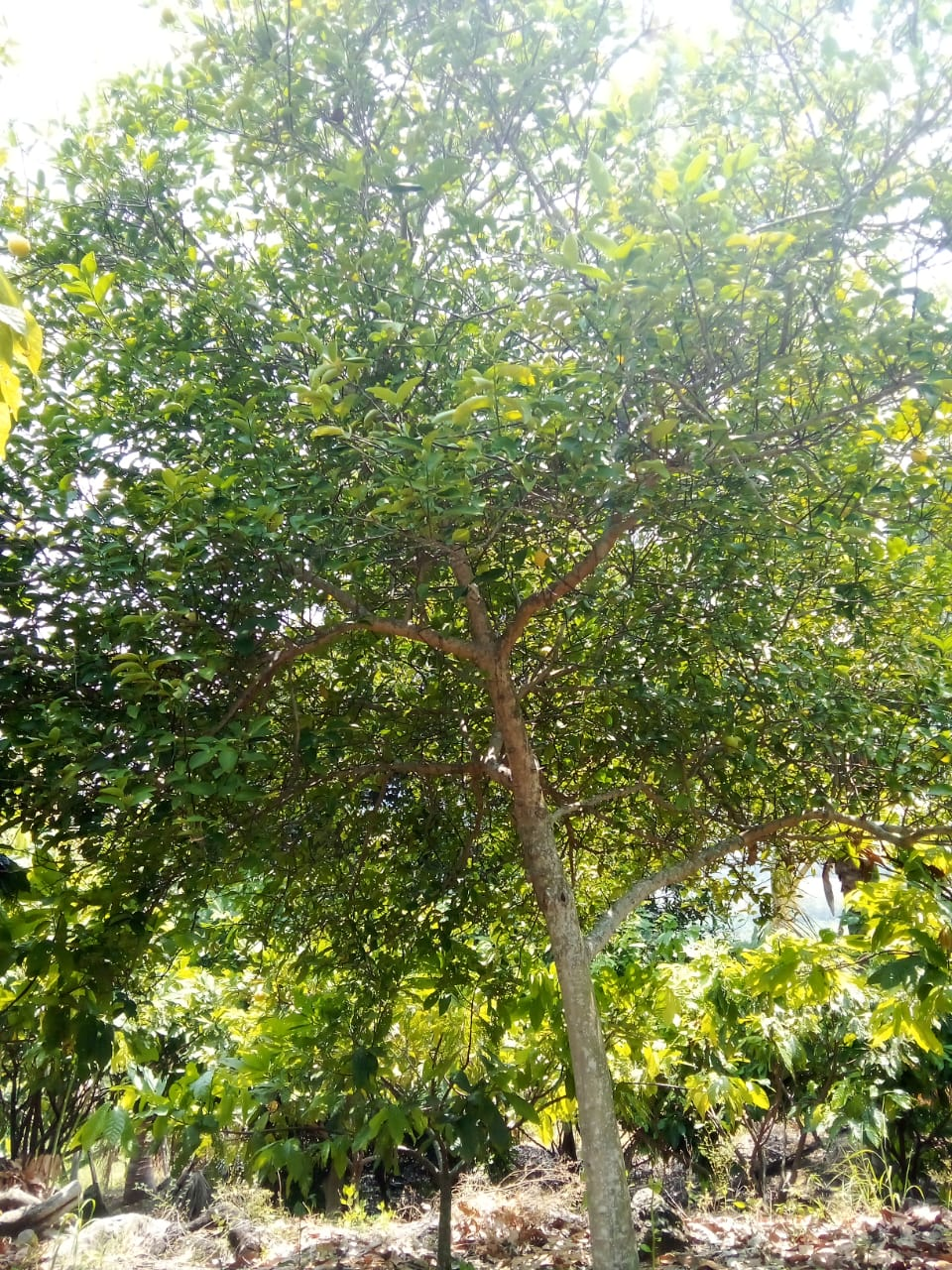


Figure S1 (b). *Citrus limetta sp* tree (sweet lemon)

Location: Rodríguez de Mendoza (San Nicolas, 1616 m.a.s.l., 6°18′00″S, 77°23′00″W)


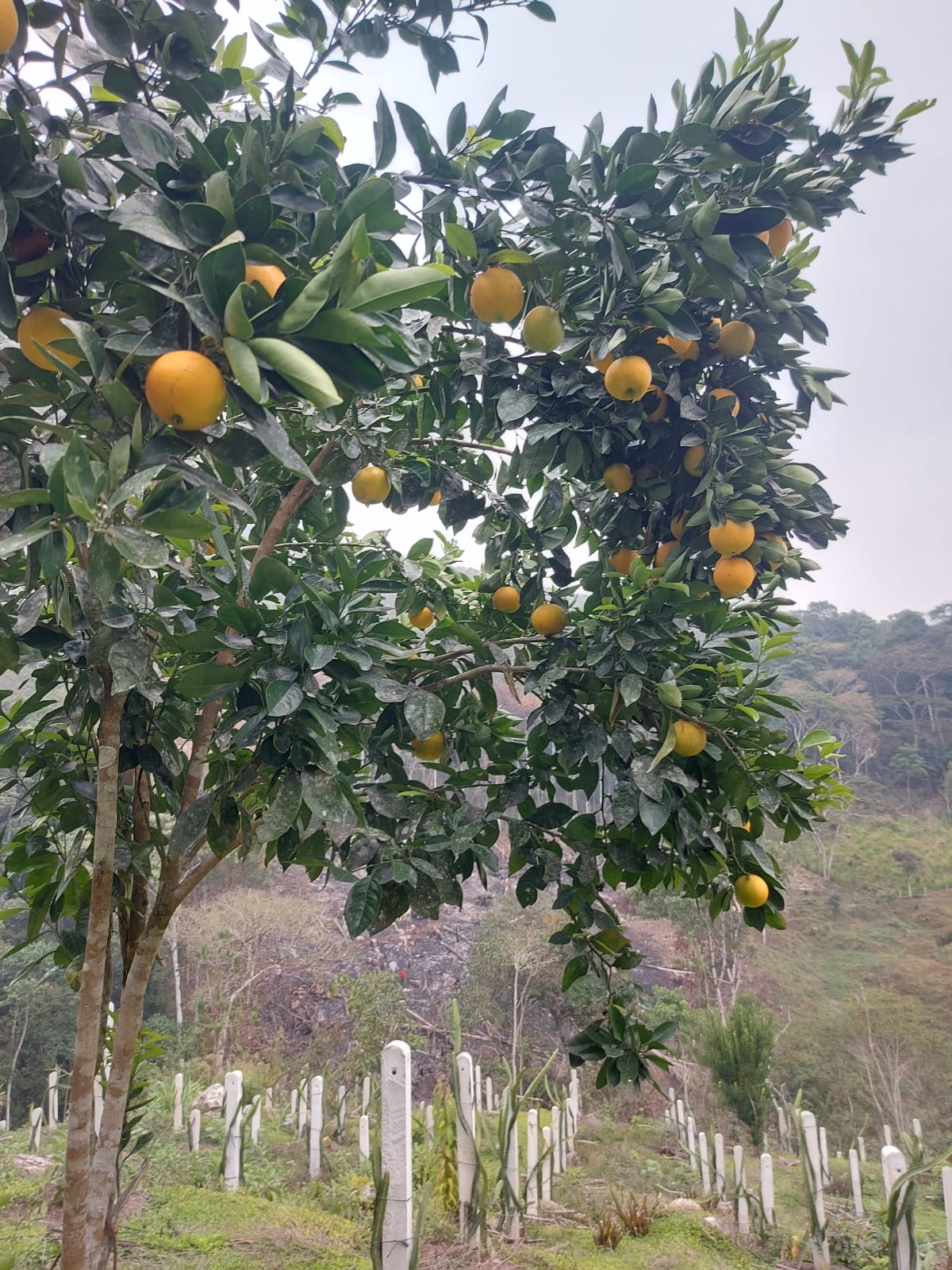


Figure S1 (c). *Citrus sinensis* (orange) tree

Location: Utcubamba (Jamalca; 1184 m.a.s.l.; 5° 53' 39'' S; 78° 14' 18'' W)


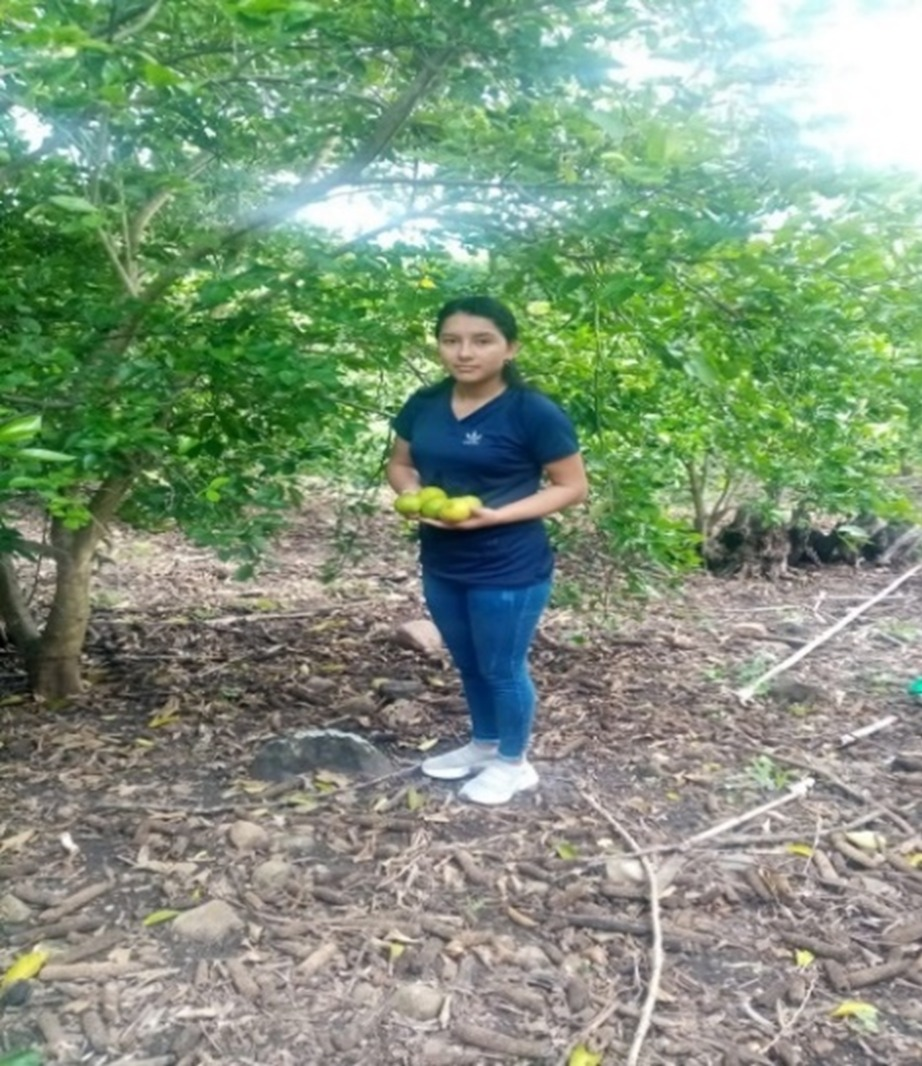


Figure S1 (d). *Citrus limetta* Risso tree (lime) tree

Location: Utcubamba (Jamalca; 1184 m.a.s.l.; 5° 53' 39'' S; 78° 14' 18'' W)
