# Supplementary material for: Exploring chemical properties of essential oils from citrus peels using green solvent
Source: Heliyon. 2024 Nov 3;10(21):e40088. doi: 10.1016/j.heliyon.2024.e40088 (PMC11570516; doi:10.1016/j.heliyon.2024.e40088)
Supplement: Multimedia component 2 [file mmc2.docx]

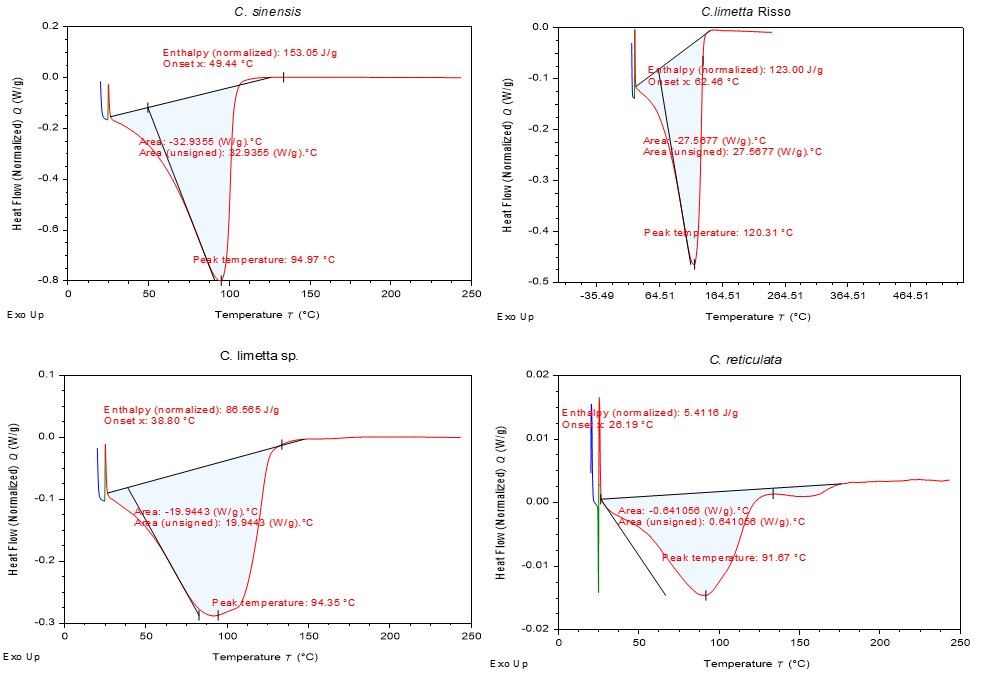


Figure S2. Thermal properties of essential oils obtained from citrus peels

*Exo Up:* Exothermic transitions up
