# Supplementary material for: Exploring chemical properties of essential oils from citrus peels using green solvent
Source: Heliyon. 2024 Nov 3;10(21):e40088. doi: 10.1016/j.heliyon.2024.e40088 (PMC11570516; doi:10.1016/j.heliyon.2024.e40088)
Supplement: Multimedia component 3 [file mmc3.docx]

- *C. sinensis*_03-200 to 3200
- *C. limetta* sp._03-200 to 3200
- *C. reticulata_*03-200 to 3200
- *C. limetta* Risso_03-200 to 3200

**Figure S3.1.** Characterization of essential oils from Citrus species by Raman spectroscopy


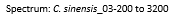

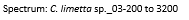

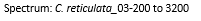

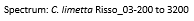


Figure S3.2. Fingerprint Raman spectra of the four essential oils: *C. sinensis*, *C. Limetta* sp., *C. reticulata* and *C. limetta* Risso.
